# Heterogeneity in the evaluation of suspected MIS-C: a cross-sectional vignette-based survey

**DOI:** 10.1186/s12887-022-03446-4

**Published:** 2022-07-04

**Authors:** Claudia A. Rosu, Anna M. Martens, Jeffrey Sumner, Eva J. Farkas, Puneeta Arya, Alexy Boudreau Arauz, Vandana L. Madhavan, Hector Chavez, Shawn D. Larson, Oluwakemi Badaki-Makun, Daniel Irimia, Lael M. Yonker

**Affiliations:** 1 Massachusetts General Hospital, Institute of Health Professionals, Boston, MA USA; 2 Harvard Medical School, Boston, MA USA; 3 Massachusetts General Hospital, 55 Fruit Street, Boston, MA 02114 USA; 4 Massachusetts General Hospital, Mucosal Immunology and Biology Research Center, Boston, MA USA; 5 Department of Pediatric Emergency Medicine, Holtz’s Children’s Hospital, Miami, FL USA; 6 Department of Surgery, University of Florida, Gainesville, FL USA; 7 Department of Pediatrics, Johns Hopkins Hospital, Baltimore, MD USA; 8 Department of Surgery, Massachusetts General Hospital, Center for Engineering in Medicine, Boston, MA USA; 9 Shriners Burn Hospital, Boston, MA USA

**Keywords:** Pediatric COVID-19, Multisystem inflammatory syndrome in children

## Abstract

**Background and Objectives:**

Multisystem Inflammatory Syndrome in Children (MIS-C) is an emerging complication of COVID-19 which lacks a definitive diagnostic test and evidence-based guidelines for workup. We sought to assess practitioners' preferences when initiating a workup for pediatric patients presenting with symptoms concerning for MIS-C.

**Methods:**

In a cross-sectional vignette-based survey, providers were presented with clinical vignettes of a patient presenting with 24 h of fever from a community with high rates of COVID-19. Respondents were asked about their general practices in pursuing a workup for potential MIS-C including testing obtained, criteria for diagnosis, and timing to confirm or rule out the diagnosis.

**Results:**

Most of the 174 respondents were physicians from the United States at academic medical centers. The majority of providers would not initiate MIS-C workup for fever and non-specific symptoms unless the fever lasted more than 72 h. Skin rash, abdominal pain, and shortness of breath were symptoms that raised greatest concern for MIS-C. Most providers would obtain COVID-19 PCR or antigen testing, plus blood work, in the initial workup. The list of laboratory studies providers would obtain is extensive. Providers primarily rely on cardiac involvement to confirm a MIS-C diagnosis, and establishing a diagnosis takes 24–48 h.

**Conclusions:**

Significant heterogeneity exists amongst providers as to when to initiate the MIS-C workup, the order and content of the workup, and how to definitively diagnose MIS-C. A diagnostic test with high sensitivity and specificity for MIS-C and refined evidence-based guidelines are needed to expedite diagnosis and treatment.

## Background

Multisystem Inflammatory Syndrome in Children (MIS-C) is an emerging, rare but severe complication of COVID-19 that can result in life-threatening multisystem organ failure and death. MIS-C develops weeks to months following infection with SARS-CoV-2 and presents with unremitting fever and a constellation of symptoms including rash, gastrointestinal symptoms, edema of the hands/feet, oral mucosal changes, conjunctivitis, lymphadenopathy, and neurological symptoms [1, 2]. Cardiac involvement is common; almost half of patients require vasopressor support and 80% require intensive care [3]. Because of the range of clinical presentations and risk for rapid progression, early diagnosis and treatment are key [1, 2]. The pathophysiology of MIS-C is not well understood, but SARS-CoV-2 antigenemia from gastric sources triggering a superantigen-like hyperinflammation post-viral inflammatory process is suggested [4–6]. The criteria for MIS-C diagnosis established by the Center for Disease Control include patients less than 21 years old presenting with fever (> 38.0℃) for more than 24 h, laboratory evidence of inflammation and multisystem involvement, who require hospitalization, without an alternative diagnosis, and with known infection or exposure to COVID-19 within the four weeks prior to the onset of symptoms [1]. Currently, no definitive diagnostic test for MIS-C exists.

Guidelines have evolved to aid in the diagnosis of MIS-C, but existing laboratory tests lack specificity. Several professional associations have put forward guidelines for a MIS-C diagnostic approach, including the American College of Rheumatology [7] and the American Academy of Pediatrics [8]. From these guidelines, it is recommended that patients with persistent fever and other accompanying symptoms have lab work performed to look for evidence of inflammation and assess cardiac, renal, hematologic, and hepatic function. Given the potential for rapid progression, there is a low threshold to initiate this workup. These guidelines have significantly changed the standard of care and shifted the paradigm for children who present with 1–2 days of fever. If there is no evidence of bacterial infection, providers would routinely send febrile children home with a diagnosis of a viral illness prior to the COVID-19 pandemic; however, with the current guidelines, many of these patients now have labs drawn, imaging done, and are admitted to the hospital for monitoring. The laboratory tests obtained are non-specific and difficult to interpret. While certain findings may increase a clinician's suspicion for MIS-C, such as elevated inflammatory or cardiac markers, diagnosis remains challenging, and practitioners face variable degrees of uncertainty.

Given the lack of a definitive diagnostic test for MIS-C, the spectrum of clinical features associated with the disease, and the lack of evidence-based guidelines for evaluation, we sought to understand practitioners' preferences for workup in a pediatric patient presenting with possible MIS-C through a vignette-based survey.

## Methods

### Participants

Between January–February 2021, we distributed an anonymous online survey to healthcare providers worldwide using simple random sampling to find and recruit the study participants. We recruited emergency medicine, pediatric, medicine-pediatric, family medicine, and pediatric subspecialty practitioners. Access to hospital providers' listservs from their specific departments and professional medical associations' listservs was required and granted. We invited providers to participate in the study via an email explaining the study goals. Participants were provided with an IRB-approved statement declaring that informed consent was implied by opening the survey link. The MGB Institutional Review Board approved this study (MGB IRB #2020P003972).

### Study measures

**Fig. 1:**
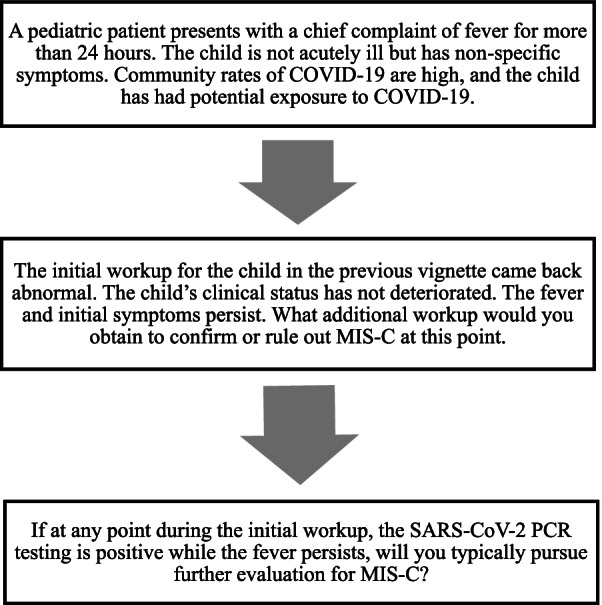
Overview of clinical vignette. The survey was based on a clinical vignette that was presented to providers in order to elicit general practices and decision-making processes when pursuing a workup for potential MIS-C

The electronic survey included three clinical vignettes, which we constructed to present a pediatric patient with fever for more than 24 h from a community with high rates of COVID-19 infection followed by various potential developments in the case (Fig. 1). The focus was on the typical approach to such a case; the timing and triggers for initiating MIS-C diagnostic workup, providers' characteristics that may influence the diagnostic approach, and the spectrum of the preferred diagnostic procedures. Existing clinical literature and questionnaires were reviewed, and expert pediatricians were consulted to support the process theoretically. This allowed the team to work backward from presenting symptoms, complaints, and practitioners' experience with suspected MIS-C cases to a controlled development of vignettes describing contextually relevant situations as the stimulus for reflection and response. Demographic items on the survey included professional role, specialty, years in practice, location, and clinical setting. Experience in treating patients with COVID-19 and MIS-C and an estimate of general patient demographics were also assessed.

### Analysis

Descriptive statistics were reported for all the questionnaire items. In addition, chi-square tests and t-tests were performed to compare the responses to the clinical vignettes across several demographic characteristics like the number of years in practice (categories ranging from 0–5, 6–10, 10–25, and over 25 years), type of clinical settings (academic medical center, community hospital, and private practice), and geographic area. For the “check all that apply” survey items, we calculated the relative percentages or the prevalence of each option among the choices selected. The data were analyzed using IBM SPSS Statistics (Version 28) predictive analytics software.

## Results

### Survey participants

**Fig. 2:**
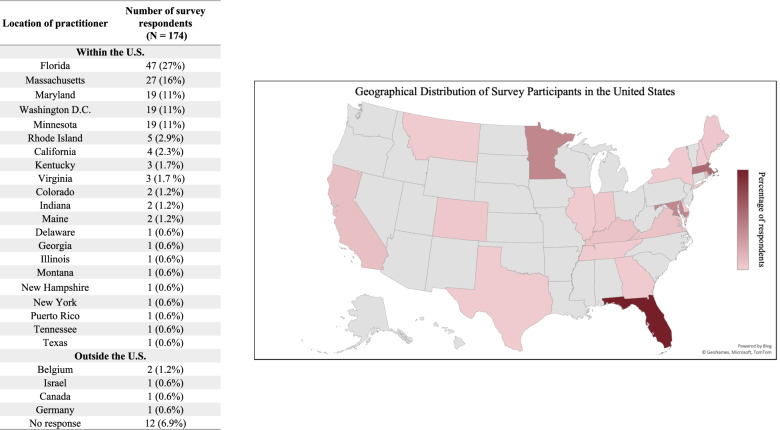
Geographical distribution of respondents within the United States. Number of survey respondents are listed by location within the United States (left); relative distribution is displayed (right). Two respondents reported practicing in more than one state; five reported working outside of the United States

A total of 174 health care providers completed the survey. Table 1 provides a summary of our sample characteristics of survey respondents. The majority of respondents were physicians (*n* = 165, 95%) from the United States (*n* = 157, 90.2%); Fig. 2 displays the distribution of respondents from the United States. Most were employed by academic medical centers (*n* = 115, 66.1%), and were either general pediatricians (*n* = 47, 27.0%), pediatric emergency medicine (*n* = 45, 26.3%), or general emergency medicine (*n* = 37, 21.3%) with a range of clinical experience and years in practice. Table 2 highlights the characteristics of the providers’ patient panel. The median estimated number of pediatric COVID-19 and MIS-C patients that providers had cared for were 34.5 and 10 over a 10-month period, respectively. Providers reported that most patients evaluated for MIS-C (median 60%, interquartile range [IQR] 30) were on Medicaid, and half of the patients were from racial/ethnic minorities.

**Table 1:** Demographics of survey respondents

**Characteristic**		***N*(%),*N*** **= 174**
**Professional Role**	MD/DO	165 (95%)
	Nurse Practitioner	3(1.7%)
	Resident	2 (1.1%)
**Specialty**	Emergency medicine Pediatrics Medicine-pediatrics Family medicine Hospital medicine Pediatric subspecialty Pediatric emergency Pediatric critical care Other	37 (21.3%) 47 (27.0%) 4 (2.3%) 1 (0.6%) 4 (2.3%) 68 (39.1%) 45 (26.3%) 6 (3.5%) 16 (9.6%)
**Years in practice**	1–5 years 5–10 years 10–25 years > 25 years	56 (32.2%) 35 (20.1%) 54 (31.0%) 24 (13.9%)
**Location**	United States DC FL ML MA Other U.S Outside the U.S.^a^	157 (90.2%) 19 (10.9%) 47 (26.8%) 19 (10.8%) 27 (15.5%) 47 (26.8%) 5 (3.0%)
**Clinical settings**	Academic medical center Urban medical center Private practice Community hospital Other	115 (66.1%) 10 (5.7%) 12 (6.9%) 26 (14.9%) 7 (4.1%)

^a^Belgium *N* = 2; Canada *N* = 1; Germany *N* = 1; Israel *N* = 1

**Table 2:** Characteristics of providers’ patient panel

**Characteristic**		**Median (IQR**^a^)
Estimated no. of pediatric COVID-19 patients seen since March 2020		34.5 (31.25)
Estimated no. of patients evaluated for MIS-C since March 2020		10.0 (16.0)
Percentage racial/ethnic minority pediatric patients		50 (40)
Percentage pediatric patients with Medicaid		60 (30)

^a^*IQR* Interquartile range

### Indications for initiating MIS-C workup

**Fig. 3:**
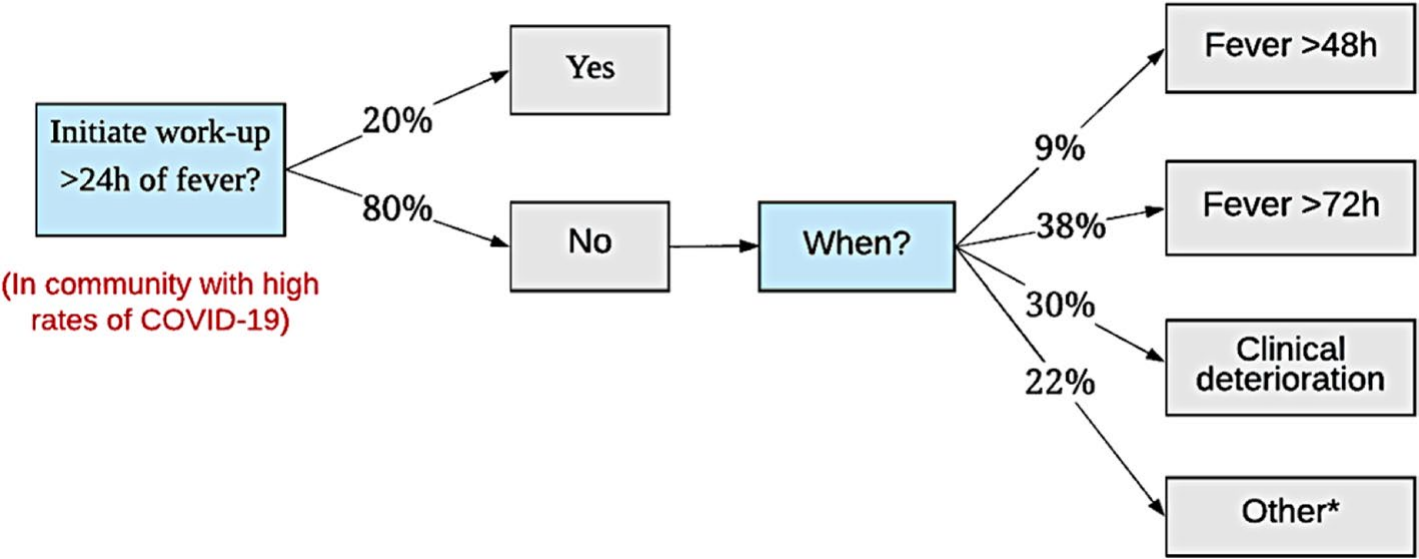
Providers Preferences for Initiating Workup. Providers were presented a clinical vignette of a non-acutely ill child presenting with fever for > 24 h and non-specific symptoms. This figure demonstrates providers’ threshold for initiating workup. *Other reasons for initiating workup: fever plus gastrointestinal or respiratory symptoms, Kawasaki-like disease or shock; clinical deterioration

Of 174 respondents, only 35 (20%) would initiate workup (blood work, imaging, or consults) for MIS-C in a non-acutely ill child presenting with fever for more than 24 h and non-specific symptoms from a community with high rates of COVID-19 (Fig. 3). Amongst the 139 providers who initially deferred a MIS-C workup, 13 (9%) would initiate a workup if the fever persisted more than 48 h, 53 (38%) would initiate the workup if the fever persisted for more than 72 h, and 42 (30%) would initiate the workup only if the child clinically deteriorates (Fig. 3).

The decision to defer MIS-C workup in a child with 24 h of fever was not associated with medical specialty (emergency medicine vs. pediatrics vs. pediatric subspecialty), years in practice (less than ten years vs. more than ten years), or clinical settings (academic medical center vs. others) (χ^2^, not significant). On average, the practitioners who chose to defer the MIS-C workup had seen significantly more COVID-19 cases (mean = 43.5, standard error of the mean [SEM] = 2.67) than those who chose to initiate workup for the same child; (mean = 29.4, SEM = 3.37; t-test, *p* = 0.01). Importantly, this finding had a large effect size (d = 0.76) suggesting that from a population perspective, the more acute pediatric COVID-19 cases that are seen, the more likely MIS-C evaluations could be delayed. There was no statistically significant difference in initiating MIS-C workup for the child described above by the number of patients the providers had previously evaluated for MIS-C, as assessed by an independent-samples t-test.

### Clinical factors impacting workup preferences for MIS-C

**Fig. 4:**
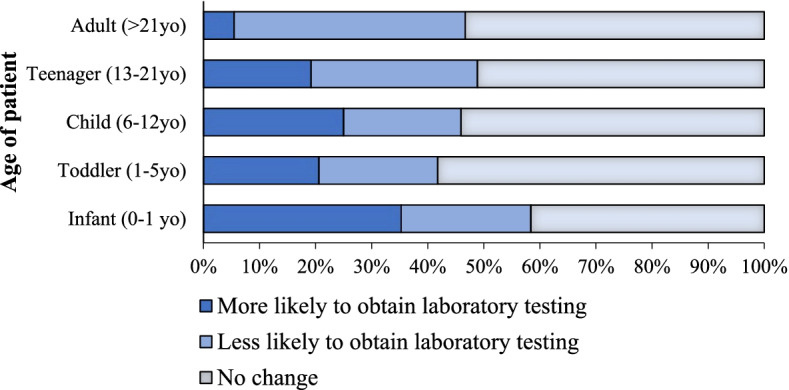
Providers’ Preferred Workup Based on Patient’s Age. Providers were asked how the initial workup would differ in a patient with concern for MIS-C based on their age

We then sought to determine patient-specific differences that would affect practitioners’ preferences in the MIS-C workup. First, we assessed how the workup would differ based on the child's age. For younger children, practitioners were more likely to obtain blood work than older children, who were more likely to be watched clinically (Fig. 4).

**Fig. 5:**
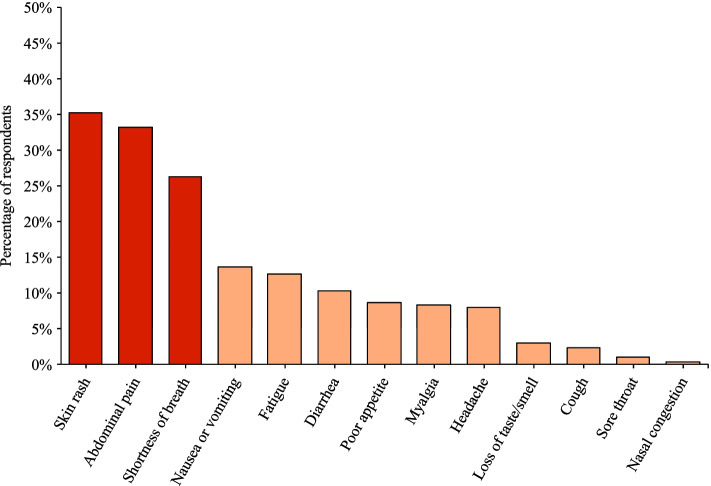
Providers’ perceptions of the most concerning symptoms for MIS-C. Providers ranked the top three presenting features, besides fever, that would make them most concerned about MIS-C in a pediatric patient with fever for more than 48 h. This chart demonstrates the most commonly ranked symptoms, with the top 3 highlighted in red

Providers were then asked to rank the top three presenting features, besides fever, that would make them most concerned about MIS-C in a pediatric patient with persistent fever for more than 48 h. Skin rash (*n* = 61, 35%), abdominal pain (*n* = 58, 33%), and shortness of breath (*n* = 46, 26%) were the most cited symptoms that raised provider concern for MIS-C (Fig. 5).

### Decision-making in the evaluation of MIS-C

**Fig. 6:**
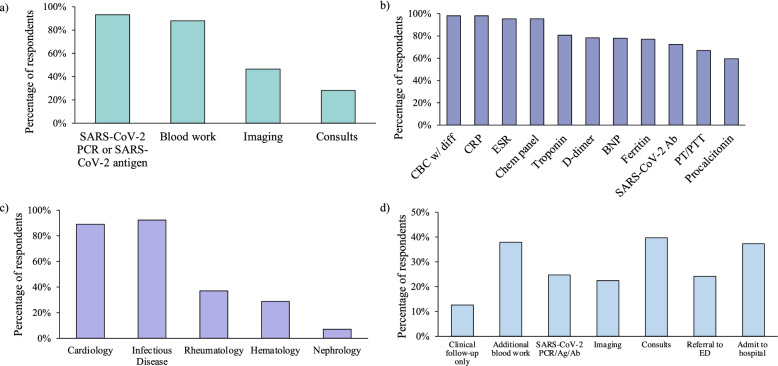
Characteristics of initial workup. **a** Initial components of workup obtained **b** Most frequently obtained laboratory tests **c** Most frequently obtained consults **d** Next steps if initial workup was abnormal. Providers were allowed to select more than one answer within each respected question

Providers were prompted about their preferences on the initial workup they would pursue for MIS-C in the pediatric patient described using multiple-choice questions in which multiple answers could be selected. Ninety-three percent (*n* = 161) of providers would obtain COVID-19 antigen or PCR testing, 88% (*n* = 153) would obtain blood work, 47% (n = 81) would obtain imaging, and 28% (*n* = 49) would obtain subspecialty consults (Fig. 6a).

Of the providers who would obtain blood work, a total of 8 tests were recommended on average (SD 3.7). Of these, CBC, CRP, ESR, and chemistry panel were the most commonly obtained tests (Fig. 6b). Seventy-three percent (*n* = 94) of providers would obtain SARS-CoV-2 antibody. Amongst the 21 providers who would not order blood work initially, 20 practitioners felt that it had limited utility at this point, while 1 practitioner thought that the discomfort to the child involved in blood work outweighed any potential benefits.

If they opted to obtain imaging, 77% (*n* = 61) of practitioners would order a chest x-ray as part of the initial evaluation of the child in our original vignette, while 88%(*n* = 65) would order ECG and 70% (*n* = 60) would order echocardiography. Nineteen percent (*n* = 14) of providers obtaining imaging would order an abdominal ultrasound, while 16% (*n* = 12) would prefer a chest CT and 15% (*n* = 11) an abdominal CT.

Of the providers who would seek out additional subspecialty consultation, the most requested consultants were infectious disease (*n* = 48, 92%) and cardiology (*n* = 49, 89%) (Fig. 6c).

The next steps that providers would take if the initial workup returned abnormal varied, but most commonly providers would obtain consults (*n* = 69, 40%), admit to the hospital (*n* = 65, 37%), and obtain additional lab work (*n* = 66, 38%) (Fig. 6d).

### Diagnosing MIS-C

**Fig. 7:**
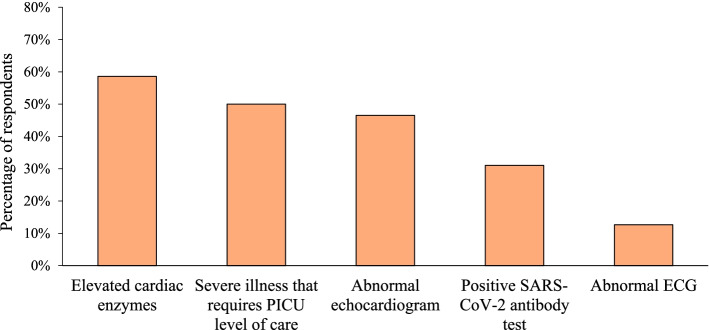
Signs and symptoms most likely to confirm a diagnosis of MIS-C. Providers’ perceptions of the signs and symptoms that were most likely to confirm the diagnosis of MIS-C. Providers were allowed to select more than one answer

We then assessed the diagnostic findings that would help providers confirm the diagnosis of MIS-C using a multiple-choice question in which multiple answers could be selected. Of the 174 respondents, the most common findings that providers felt would confirm MIS-C diagnosis were elevated cardiac enzymes (*n* = 102, 59%), severe illness requiring admission to a pediatric intensive care unit (*n* = 87, 50%), or abnormal echocardiogram (*n* = 81, 47%) (Fig. 7).

We also assessed participants' perceptions about the role of a positive COVID-19 PCR test in evaluation for MIS-C diagnosis in a child with persistent fever. One hundred and twenty-four respondents (73%) would further evaluate for MIS-C, while 46 (27%) would only follow the child clinically. In this question, providers were asked to select one option. Reasons given for opting to follow the child clinically without further workup in the setting of a positive COVID-19 PCR included the perception that a diagnosis of acute COVID-19 infection is more likely (*n* = 31, 67%), the need for additional criteria to be met, including positive SARS-CoV-2 serology (*n* = 9, 10%), or a decline in child's clinical status (*n* = 25, 54%) (providers were allowed to select multiple options).

### Time to diagnosis

**Fig. 8:**
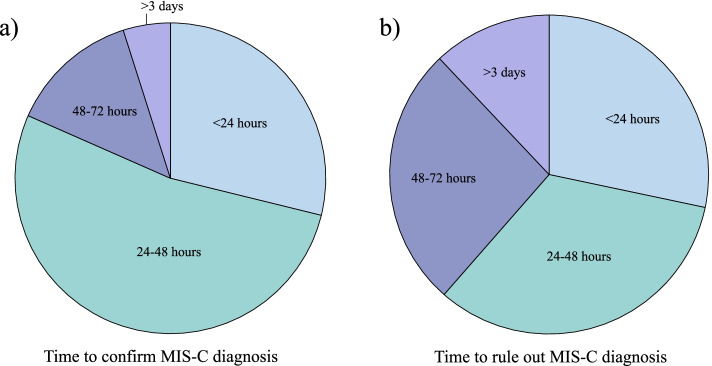
Time to confirm or rule out MIS-C. **a** Average time it takes to confirm MIS-C diagnosis based on providers’ practice setting. **b** Average time it takes to rule out MIS-C diagnosis based on provider’s setting

Finally, we wanted to ascertain how long it typically takes to confirm or exclude a MIS-C diagnosis based on our study respondents' experience at their workplace. The majority of participants reported that it took 24–48 h to confirm or rule out MIS-C (Fig. 8a and b, respectively). There was no statistically significant association between the time to confirm or exclude a diagnosis of MIS-C and the type of clinical setting (academic medical center vs. urban medical center/community hospital vs. private practice/other settings), highlighting the high degree of ambiguity in current diagnostic guidelines, the high costs associated with evaluating MIS-C, and urgent need to establish improved diagnostic criteria for MIS-C across a range of clinical settings.

## Discussion

MIS-C is a rare but severe sequela of COVID-19 in children; early diagnosis and treatment are critical to reduce or avoid MIS-C morbidity and mortality. Unfortunately, the number of pediatric illnesses that present with similar symptoms is broad, making it challenging to differentiate MIS-C from other more benign childhood illnesses, as well as distinguish it from bacterial infections and Kawasaki disease. Because of the non-specific presenting symptoms, potential for rapid progression, and the life-threatening nature of MIS-C, there is a low threshold for initiating workup. Our study shows significant heterogeneity amongst providers as to when they initiate MIS-C workup, components of the workup, and how to confirm MIS-C diagnosis.

Given how extensive the MIS-C workup is, it is important to carefully consider which patients warrant an evaluation. Current guidelines, however, lack explicit screening criteria. While the CDC case definition of MIS-C includes fever for at least 24 h, guidelines are unclear on the duration of fever that should generate a workup. This lack of clarity is reflected in our study, where providers were divided when asked about the duration of fever that would prompt them to initiate workup. Additional thought also must be put into excluding other febrile illnesses, such as viral illnesses, bacterial infections, or Kawasaki disease, which can be particularly challenging given the overlapping clinical features [11].

Providers who had seen more COVID-19 cases were more likely to initiate MIS-C workup, suggesting that the more exposure providers have to COVID-19, the more likely they are to consider MIS-C. Our study also shows that providers are more likely to initiate a workup in younger children than in older children; however, current guidelines do not differ based on age. Such heterogeneity could lead to missed or delayed diagnosis.

Once it is determined that a child warrants workup for MIS-C, there is a recommended set of “Tier 1” screening labs, which if abnormal, prompt additional evaluation, including “Tier 2” labs, EKG, and echocardiogram [7]. Both tiers of labs are non-specific and their ability to distinguish MIS-C from other causes of acute febrile illnesses. Our study shows that providers often do obtain lab work, which is consistent with the existing guidelines, but it is unclear how these results help guide diagnosis. For example, the majority of providers would obtain SARS-CoV-2 serology. However, with over 13 million children under 18 years old testing positive for COVID-19 thus far in the pandemic in the United States alone, we can expect that most children will develop post-vaccination and/or post-infectious antibodies at some point. Thus, relying on serology as a laboratory marker will become even less specific [9]. Additional workup that providers obtained beyond laboratory work, including consults and imaging, was largely heterogeneous and inconsistent.

Once a patient is diagnosed with MIS-C, they are usually treated with IVIG and/or systemic steroids, which are thought to improve clinical outcomes. The most common findings that our respondents perceived to confirm MIS-C diagnosis were elevated cardiac enzymes, abnormal echocardiogram, or severe illness requiring admission to a pediatric intensive care unit. While cardiac involvement is one of the most feared complications of MIS-C and is present in up to 80% of cases, it is not necessary to diagnose MIS-C [10]. Therefore, heavy reliance on cardiac findings to confirm MIS-C diagnosis could mean that providers are missing or under-reporting cases of MIS-C. This highlights the need for more clear diagnostic criteria.

Limitations of this study include it being a cross-sectional survey of self-reported preferences in providers, with potential for sampling and response biases. We did not examine the medical records to compare with real observed cases to ascertain accuracy. Our sample was skewed toward providers from academic medical centers where access to laboratory tests, imaging, and specialized consultants is readily available. Further research is needed to assess what happens in other clinical settings (e.g., rural primary care offices) where access to testing, imaging, and specialized consultants would be limited. Identifying MIS-C and differentiating it from other illness is an important step in the diagnostic process. While our study focused on the process of identifying MIS-C, we did not ask providers to specify how they would exclude other illnesses, such as bacterial infections. However, this study highlights the need to better understand the initial presentation that should prompt further diagnosis, as well as a simplified diagnostic test that is more specific and widely accessible to help facilitate timely diagnosis and treatment.

## Conclusion

Our study illustrates significant heterogeneity among healthcare providers as to when to initiate MIS-C workup, the order and context of the workup, and the criteria for MIS-C diagnosis. The current guidelines are designed to be sensitive as to minimize missed or delayed diagnoses. However, this subsequently results in high healthcare resource utilization. A diagnostic test is needed that is highly sensitive and specific in order to expedite diagnosis and treatment. With the United States in its fourth wave of the COVID-19 pandemic, it is evident that SARS-CoV-2 and MIS-C will likely remain a threat for the foreseeable future. The scientific community therefore needs to urgently build on existing research and refine clinical guidelines to minimize pediatric MIS-C morbidity and mortality.

## Data Availability

The datasets generated and/or analyzed during the current study are not publicly available due Mass General Brigham policies. However, data used in this study may become available to other researchers upon reasonable request to the corresponding author and in compliance with the Mass General Brigham Innovations Office.

## Abbreviations

MIS-C: Multisystem inflammatory syndrome in children
SEM: Standard error of the mean
IQR: Interquartile range
